# Spot Urine Formulas to Estimate 24-Hour Urinary Sodium Excretion Alter the Dietary Sodium and Blood Pressure Relationship

**DOI:** 10.1161/HYPERTENSIONAHA.120.16651

**Published:** 2021-04-05

**Authors:** Abu Mohd Naser, Feng J. He, Mahbubur Rahman, Norm R.C. Campbell

**Affiliations:** 1 Hubert Department of Global Health, Emory Global Diabetes Research Center (A.M.N.), Rollins School of Public Health, Emory University, Atlanta, GA.; 2 Department of Epidemiology (A.M.N.), Rollins School of Public Health, Emory University, Atlanta, GA.; 3 Centre for Environmental and Preventive Medicine, Wolfson Institute of Preventive Medicine, Barts and The London School of Medicine and Dentistry, Queen Mary University of London, United Kingdom (F.J.H.).; 4 Environmental Interventions Unit, Infectious Diseases Division, International Center for Diarrhoeal Disease Research, Bangladesh, India (M.R.).; 5 Department of Medicine, O’Brien Institute of Public Health, Libin Cardiovascular Institute of Alberta at the University of Calgary, Canada (N.R.C.C.).

**Keywords:** blood pressure, creatinine, minerals, potassium, sodium

## Abstract

We evaluated the relationship between estimated 24-hour urinary sodium excretion from the Kawasaki, Tanaka, and INTERSALT (International Study of Sodium, Potassium, and Blood Pressure) formulas and blood pressure (BP). We pooled 10 034 person-visit data from 3 cohort studies in Bangladesh that had measured 24-hour urine sodium (m-24hUNa), potassium, creatinine excretion, and BP. We used m-24hUNa, potassium, and creatinine where necessary, rather than spot urine values in the formulas. Bland-Altman plots were used to determine the bias associated with formula-estimated sodium relative to m-24hUNa. We compared the sodium excretion and BP relationships from m-24hUNa versus formula-estimated sodium excretions, using restricted cubic spline plots for adjusted multilevel linear models. All formulas overestimated 24-hour sodium at lower levels but underestimated 24-hour sodium at higher levels. There was a linear relationship between m-24hUNa excretion and systolic BP, while estimated sodium excretion from all 3 formulas had a J-shaped relationship with systolic BP. The relationships between urine sodium excretion and diastolic BP were more complex but were also altered by using formulas. All formulas had associations with BP when a sex-specific constant sodium concentration was inserted in place of measured sodium. Since we used the m-24hUNa, potassium, and creatinine concentrations in formulas, the J-shaped relationships are due to intrinsic problems in the formulas, not due to spot urine sampling. Formula-estimated 24-hour urine sodium excretion should not be used to examine the relationship between sodium excretion and BP since they alter the real associations.

## Abstract 2

Supplemental Digital Content is available in the text.

**See Editorial, pp 2138–2139**

High sodium intake is a major dietary risk factor for raised blood pressure (BP) and a risk for cardiovascular disease.^1,2^ However, there is a lack of clarity regarding the health effects of sodium intake below 87 mmol (2000 mg)/day or salt (sodium chloride) intake below 5 g/day. High-quality epidemiological and clinical studies that use 24-hour urine sodium excretion as a proxy of sodium intake have shown a positive linear association between sodium intake and BP,^3–5^ as well as cardiovascular disease events and total mortality.^6–8^ However, observational studies that used spot urine sodium excretion as a proxy of sodium intake have reported J- or U-shaped relationships with cardiovascular disease,^9^ suggesting lower sodium intake is harmful. Such findings are inconsistent with the World Health Organization and the National Academies of Science, Engineering, and Medicine reviews of the evidence and recommendations to lower sodium intake below 87 mmol (2000 mg)/day and 100 mmol (2300 mg/day, respectively).^10,11^

One potential reason for controversy regarding lower sodium intake is the use of spot urine samples with estimating equations to predict the daily sodium intake in epidemiological studies.^3^ Although 24-hour urinary sodium excretion is a better proxy of sodium intake than that estimated from spot urine equations,^3^ collection of spot urine is less expensive, more feasible, and convenient.^11–13^ Formulas are used to estimate the daily urine sodium from spot urine concentrations. Currently, INTERSALT,^14^ Kawasaki,^15^ and Tanaka^16^ formulas are commonly used to predict 24-hour urine sodium excretion from spot urine samples (Table S1 in the Data Supplement). All three formulas use age, height, weight, spot urine sodium, creatinine concentrations, and some constants. INTERSALT and Kawasaki formula are sex specific, and INTERSALT formula includes spot urine potassium.

We hypothesized that the J- or U-shaped relationships are due to inaccurate estimation of sodium excretion by spot urine formulas.^3,17^ There are inherent problems within the formulas because they use age, sex, height, creatinine, and weight, all of which are predictors of cardiovascular disease. We used measured 24-hour urinary sodium (m-24hUNa) concentrations in the spot urine formulas, rather than spot urine values, to evaluate the sodium-BP relationship from formula-estimated 24-hour urine sodium compared with m-24hUNa. Our overarching objective was to evaluate whether the sodium-BP relationship was changed due to inherent problems within the formulas.

## Methods

The data that support the findings of this study are available from the corresponding author upon reasonable request.

### Data Sources

We analyzed the urinary sodium excretion and BP relationship using pooled data from 3 longitudinal studies from Bangladesh. The longitudinal studies were conducted in southwest coastal Bangladesh (Figure S1) by the International Centre for Diarrhoeal Disease Research, Bangladesh. This region has seawater as a source of sodium intake.^18,19^ We pooled 10 034 person-visit data from the 3 studies. The first cohort study followed up 383 participants for 2 visits (742 person-visits) from southwest coastal Bangladesh.^20^ The second study was a population-based stepped-wedge randomized controlled trial in southwest coastal Bangladesh that followed up 1190 participants for 5 monthly visits (5745 person-visits) to investigate the health effects of providing access to low-salinity groundwater.^21,22^ The third cohort study followed up 293 participants from southwest coastal and 277 from noncoastal central Bangladesh for 7 visits (3547 person-visit data: 1773 from coastal and 1774 from noncoastal regions).

### BP Measurement

We measured the participants’ BP between 7 AM and 2 PM in all visits using an accuracy validated Omron HEM–907 (Kyoto, Japan) digital monitor.^23^ Participants did not consume caffeine (eg, tea and coffee) or food, did not smoke, or did not perform heavy physical activities 30 minutes before BP measurement. An appropriate-sized cuff was used to measure participants’ BP (a small-size cuff if mid-arm circumference was <22 cm, a medium-size cuff if mid-arm circumference was ≥22 to <32 cm, and a large-size cuff if mid-arm circumference was ≥32 cm). Participants rested for at least 5 minutes in a chair with back support in the sitting position with the arm supported at heart level. BP was measured 3×, and the mean was used for analyses.

### Demographic, Cardiovascular Risk Factor, and Physical Measures Data

We collected participants’ demographics (age, sex, and religion) and measured their height and weight. We collected data on smoking, work-related physical activities, alcohol consumption, and sleep hours during all visits. To calculate household wealth, we collected information on ownership of refrigerator, television, mobile phones, motorcycle, bicycle, sewing machine, chair, table, wristwatch, wardrobe wooden cot, motor pump, rice husking machine, motorized rickshaw, car, and access to electricity.

### Twenty-Four–Hour Urinary Sodium, Potassium, and Creatinine

We collected participants’ 24-hour urine at all visits in the 3 studies. Each participant received a 4-L container for urine collection and a small container to transfer the voided urine to the 4-L container. Participants were instructed to discard their first-morning urine and start urine collection by transferring the second-morning void and then transferring all voids up to and including the next morning’s first void. In the first cohort study, we provided 15-mL tubes to all participants to collect the second-morning spot urine samples. Participants were instructed to transfer a portion of the second-morning void to the 15-mL tube and the 4-L plastic container. Field research assistants recorded the collected urine volume and obtained a 15-mL urine sample from the 4-L container after stirring. Urine samples were transported to a field laboratory at 2 to 8° C. Direct ion-selective electrode methods were used to measure the urinary sodium and potassium with a semi-auto electrolyte analyzer (Biolyte2000; BioCare Corporation, Taiwan; coefficient of variation, ±5%). Creatinine was measured by the urine Jaffe reaction. We determined the completeness of 24-hour urine collection based on 2 creatinine-based methods: (1) creatinine index ≥0.7^12^ and (2) measured 24-hour urine creatinine (mCER) within 15% of Kawasaki-predicted daily urine creatinine excretion.^15^ The creatinine index ≥0.7 method excludes samples that have low 24-hour urinary creatinine excretion, whereas mCER within 15% of Kawasaki-predicted daily urine excretion method excludes samples that have low or high 24-hour urine creatinine excretion.^12^ The creatinine index was defined as mCER in milligrams/(21×bodyweight) for women and mCER in milligrams/(24×bodyweight) for men.^12^ Kawasaki-predicted daily urine creatinine excretion was calculated using the Kawasaki formula: −12.63×age+15.12×weight+7.39×height−79.90 for men, and −4.72×age+8.58×weight+5.09× height−74.50 for women.^24^

### Statistical Analyses

The household wealth score was derived by a principal component analysis using the household asset ownership data and categorized into wealth quintiles.^25^ The mean and SD are reported for continuous covariates and urine sodium measurements. The categorical covariates are reported as proportions.

#### Calculation of Formula-Estimated 24-Hour Sodium Excretion

For the person-visits without any self-reported missed urine voids, we inserted the m-24hUNa, potassium, and mCER in 3 formulas,^26^ to calculate the formula-estimated 24-hour urine sodium excretion (ie, the m-24hUNa, potassium, and mCER concentrations replaced the usual practice of using spot urine sodium, potassium, and creatinine concentrations, in the formulas). The rationale was that if altered sodium-BP relationships are observed for the formula-estimated sodium compared with the sodium-BP relationship for m-24hUNa, the altered relationship will be due to the formulas and not related to spot urine sampling. The same approach was used to assess the sodium excretion-mortality relationship,^17,26^ but to our best knowledge, it has not been used for assessing the sodium excretion and BP relationships.

#### Estimation of Bias Associated With Formula-Estimated 24-Hour Urine Sodium Excretion

We plotted the kernel density plots for measured versus formula-estimated 24-hour urinary sodium. The mean bias was calculated as the difference between the formula-estimated and m-24hUNa excretions. We used paired *t* tests to determine whether the mean bias was statistically significant. We then used the Bland-Altman plots to compare the formula-estimated and m-24hUNa excretion.

#### Evaluation of Urinary Sodium Excretion and BP Relationships

In the main analyses, we excluded person-visits that had any missed urine void. In 2 sensitivity analyses, we also excluded 24-hour urine samples that did not fulfill the following criteria: (1) a creatinine index ≥0.7 and (2) mCER within 15% of Kawasaki-predicted daily urine creatinine excretion. We plotted the restricted cubic spline,^27^ to examine the relationship between BP and urinary sodium excretion. We used default 4 knots at the 5th, 35th, 65th, and 95th percentiles to create the restricted cubic spline plots.^28^ Restricted cubic spline plots consider cubic polynomial segments after the first knot and before the last knot.^29^ We created the restricted cubic spline plots with the predicted BP values after running multilevel linear models with 3-level random intercepts to account for the clustering of data at the participant, household, and community level. We adjusted models for age, sex, body mass index, smoking and alcohol consumption, physical activities, religion, hours of sleep, and household wealth. Religion was considered a covariate because of differences in dietary habits between Hindus and Muslims—Hindus are often vegetarian and tend to eat less meat, but Muslims usually consume meat.^30^ Sleep hours were considered as a covariate because less sleep is associated with high BP.^31^

We also modeled urinary sodium as a categorical variable by using quartiles of urinary sodium. Similar multilevel linear models to those described above were used to determine the associations of quartile 2, 3, and 4 urinary sodium with BP compared with quartile 1. We reported findings of unadjusted models; models adjusted for age, sex, and body mass index; and models additionally adjusted for smoking and alcohol consumption, physical activity, religion, hours of sleep, and household wealth. We reported the *P* for linear trends.

Sex-specific constant mean sodium concentrations were inserted in formulas to evaluate whether the altered sodium-BP relationships are independent of the sodium concentrations in the equations after implementing similarly restricted cubic spline plots and quartile analyses described above. All analyses were conducted in STATA, version 16.0.

We conducted several sensitivity analyses. First, we created the restricted cubic spline plots among the completed 24-hour samples determined by the creatinine index ≥0.7 and mCER within 15% of Kawasaki-predicted daily urine creatinine excretion. The purpose of restricting the analyses among the complete 24-hour samples was to evaluate the urine sodium-BP relationship after excluding the samples with low or high urine creatinine excretion.

The Kawasaki and Tanaka formulas were initially developed from participants whose urine biomarkers were measured in the second-morning spot urine samples^15^; however, we inserted the 24-hour urine concentrations of biomarkers in the spot urine formula because we did not collect spot urine samples in cohort 2 and 3 studies. In the cohort 1 study, we collected second-morning urine spot samples along with 24-hour urine samples. In the second sensitivity analyses, we compared the urine sodium-BP relationship from 3 types of urine sodium using the cohort 1 data: (1) m-24hUNa, (2) formula-estimated urine sodium when 24-hour urine sodium concentrations were inserted into the formulas, and (3) formula-estimated urine sodium when second-morning spot urine sodium concentrations were inserted into the formulas.

### Ethics

Written informed consent was obtained from all participants. The study protocols were approved by the Ethical Review Committee of the International Centre for Diarrhoeal Disease Research, Bangladesh.

## Results

The main sociodemographic characteristics of the study population are in Table 1. Among the 10 034 person-visits, 61% were by women (Table 1). The mean age was 42.6 (±14.4) years, and the mean body mass index was 22.4 (±4) kg/m^2^. Of the participants, 61% reported no current or previous smoking history, 97.4% did not consume alcohol, and 37% had a sedentary work-related physical activity level (Table 1).

**Table 1.**
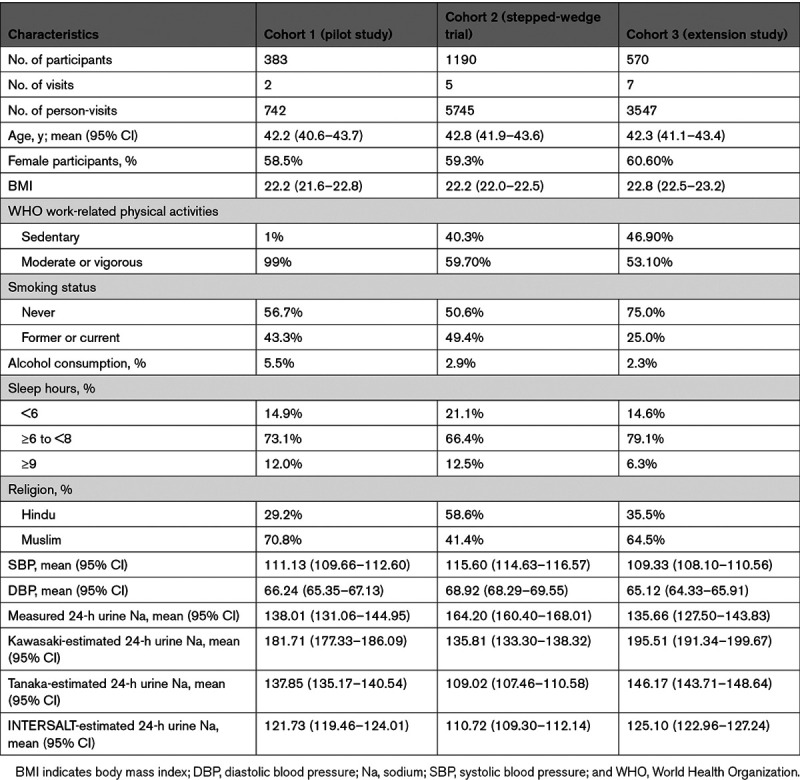
Characteristics of the Participants From 3 Studies

### Bias Associated With Formula-Estimated 24-Hour Urine Sodium Excretion

**Figure 1.**
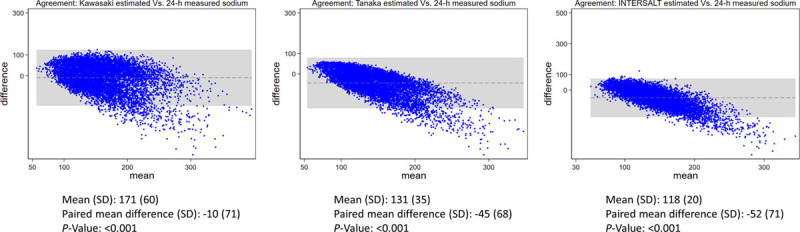
**Bland-Altman plot comparing 24-h urinary sodium excretion estimated using the 3 spot urine formulas and directly measured in the 24-h urinary collections.** The difference indicates difference between each formula-estimated 24-h urine sodium and measured 24-h sodium. Mean indicates mean of each formula-estimated 24-h urine sodium and measured 24-h sodium.

The mean m-24hUNa was 168±73 (±SD) mmol/24 hours or 3864±1679 mg/24 hours; the Kawasaki estimate was 159±47 mmol/24 hours or 3657±1081 mg/24 hours; the Tanaka estimate was 124±29 mmol/24 hours or 2852±667 mg/24 hours; and the INTERSALT estimate was 118±30 mmol/24 hours or 2714±690 mg/24 hours. All formula-estimated urinary sodium had less variability than the m-24hUNa, and the Tanaka-estimated 24-hour sodium had the least variability (Figure S2). The mean bias (ie, the difference between formula-estimated and measured 24-hour sodium) was −10 mmol/24 hours or −230 mg/24 hours for the Kawasaki-estimated urine sodium, −45 mmol/24 hours or −1035 mg/24 hours for the Tanaka-estimated urine sodium, and −52 mmol/24 hours or 1196 mg/24 hours for the INTERSALT-estimated urine sodium (Figure 1). Bland-Altman plots show that all 3 formulas overestimated 24-hour sodium at lower levels and underestimated 24-hour sodium at higher levels (Figure 1).

### Sodium Excretion and Systolic BP Relationship

**Figure 2.**
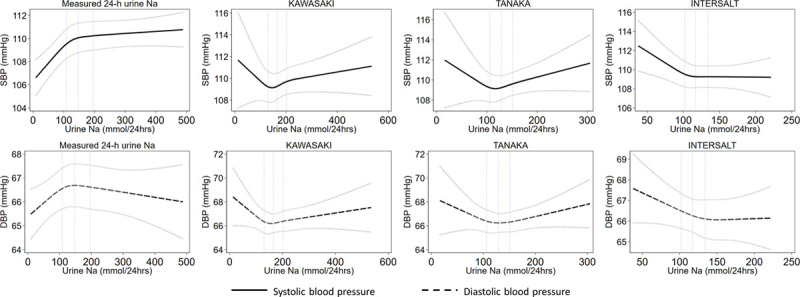
**Restricted cubic spline plots and 95% CI (dotted lines) for sodium (Na) excretion and blood pressure relationship using different methods of estimating urinary Na excretion.** Only 24-h urines with self-reported missing voids were excluded. Models were adjusted for age, sex, body mass index, smoking status, physical activity, sleep, alcohol consumption, religion, and household wealth. Vertical dotted blue lines indicate 25th, 50th, and 75th percentile distribution of urine Na excretion. DBP indicates diastolic blood pressure; and SBP, systolic blood pressure.

The restricted cubic spline plot illustrated a steep positive linear sodium-systolic BP (SBP) relationship up to 145 mmol or 3335 mg per 24 hours of m-24hUNa, followed by a less steep positive linear relationship at higher levels (Figure 2). In contrast, we found a J-shaped sodium excretion and SBP relationship for the Kawasaki-, Tanaka-, and INTERSALT-estimated urine sodium (Figure 2). All 3 estimation approaches had a negative relationship between estimated 24-hour urine sodium and SBP, almost up to the median of estimated 24-hour urine sodium (Figure 2). Compared with the quartile 1 person-visits of the m-24hUNa, those in quartile 2 had 1.46 (95% CI, 0.89–2.04) mm Hg higher SBP, those in quartile 3 had 1.55 (95% CI, 0.77–2.32) mm Hg higher SBP, and those in quartile 4 had 1.68 (95% CI, 0.98–2.37) mm Hg higher SBP in the final multivariable-adjusted models (*P* for linear trend, <0.001; Table 2). Compared with the quartile 1 person-visits of the Kawasaki-estimated 24-hour urine sodium, those in quartile 2 had −0.48 (95% CI, −1.42 to 0.47) mm Hg change in SBP, those in quartile 3 had −0.34 (95% CI, −1.16 to 0.49) mm Hg change in SBP, and those in quartile 4 had 0.43 (95% CI, −0.61 to 1.47) mm Hg change in SBP (*P* for linear trend, 0.397; Table 2). Compared with quartile 1 person-visits of the Tanaka-estimated 24-hour urine sodium, those in quartile 2 had −0.82 (95% CI, −1.71 to 0.07) mm Hg change in SBP, those in quartile 3 had −0.72 (95% CI, −1.54 to 0.11) mm Hg change in SBP, and those in quartile 4 had 0.30 (95% CI, −0.78 to 1.38) mm Hg change in SBP (*P* for linear trend, 0.552; Table 2). Compared with quartile 1 person-visits of the INTERSALT-estimated 24-hour urine sodium, those in quartile 2 had −0.50 (95% CI, −1.36 to 0.36) mm Hg change in SBP, quartile 3 had −0.48 (95% CI, −1.65 to 0.68) mm Hg change in SBP, and quartile 4 had −0.21 (95% CI, −1.64 to 1.22) mm Hg change in SBP (*P* for linear trend, 0.798; Table 2).

**Table 2.**
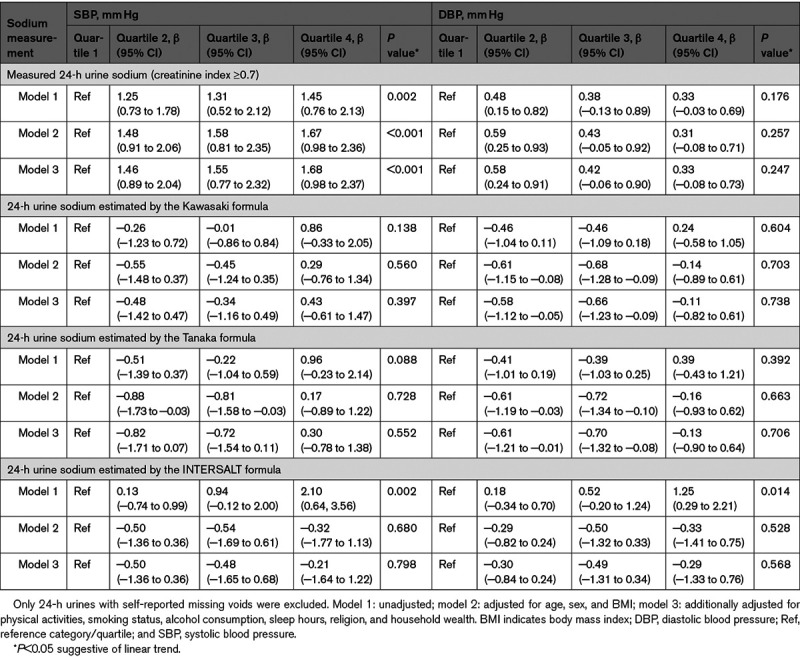
Association Between Quartiles of Urinary Sodium and Blood Pressure Relationship

**Figure 3.**
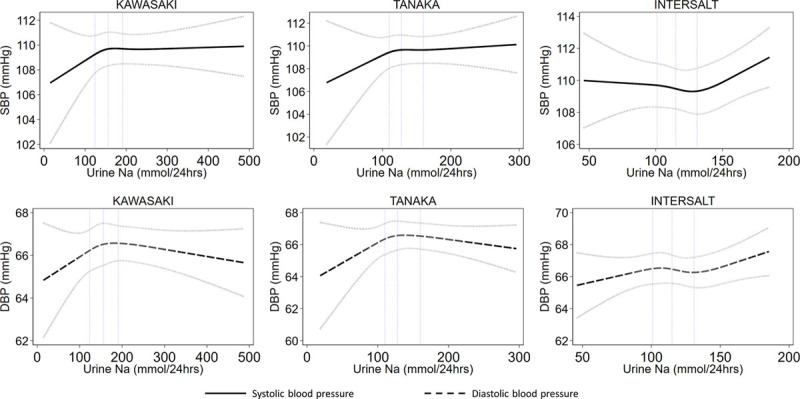
**Restricted cubic spline plots and 95% CI (dotted lines) for formula-estimated 24-h sodium (Na) excretion and blood pressure relationship, when constant urinary Na was used in formula.** Only 24-h urines with self-reported missing voids were excluded. Models were adjusted for age, sex, body mass index, smoking status, physical activity, sleep, alcohol consumption, religion, and household wealth. Vertical dotted blue lines indicate 25th, 50th, and 75th percentile distribution of urine Na excretion. DBP indicates diastolic blood pressure; Ref, reference category/quartile; and SBP, systolic blood pressure.

Inserting a sex-specific mean constant sodium concentration in the formulas resulted in a loss of all J-shaped appearances (Figure 3). When a sex-specific constant sodium value was used in the formula, compared with the quartile 1 person-visits of the Kawasaki-estimated 24-hour urine sodium, those in quartile 4 had 0.86 (95% CI, −0.20 to 1.92) mm Hg higher SBP in the final model (*P* for linear trend, 0.258; Table 3). Similarly, compared with the quartile 1 person-visits of the Tanaka-estimated 24-hour urine sodium, those in quartile 4 had 0.80 (95% CI, −0.19 to 1.80) mm Hg change in SBP (*P* for linear trend, 0.246; Table 3). Compared with the quartile 1 person-visits of the INTERSALT-estimated 24-hour urine sodium, those in quartile 4 had −0.01 (95% CI, −1.44 to 1.41) mm Hg change in SBP (*P* for linear trend, 0.923; Table 3).

**Table 3.**
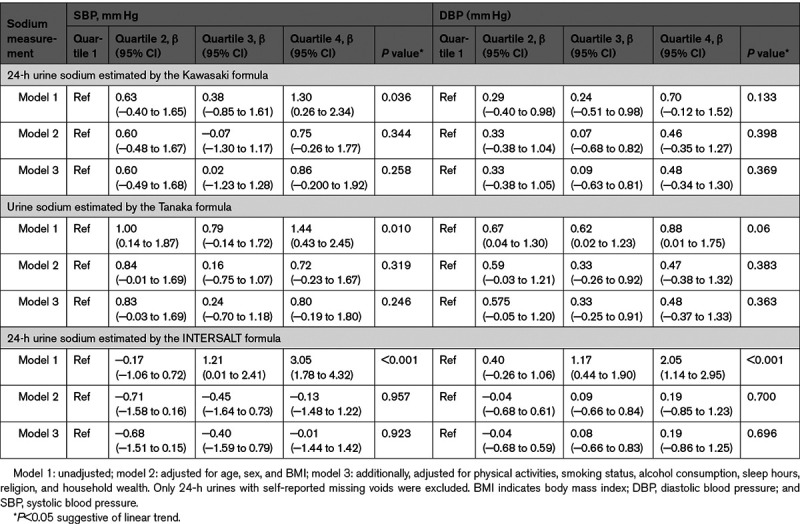
Association Between Quartiles of Formula-Estimated 24-h Urinary Sodium and Blood Pressure Relationship, When Sex-Specific Constant Urinary Sodium Was Used in Formula

### Sodium Excretion and Diastolic BP Relationship

The restricted cubic spline plot visually illustrated a steep positive linear sodium-diastolic BP (DBP) relationship up to 145 mmol or 3335 mg per 24 hours of m-24hUNa, followed by a plateaued relationship at higher levels (Figure 2). Compared with the quartile 1 person-visits of the m-24hUNa, those in quartile 4 had 0.33 (95% CI, −0.08 to 0.73) mm Hg change in DBP in the final multivariable-adjusted models (*P* for linear trend, 0.247; Table 2). Similar to SBP, we found a J-shaped sodium excretion and DBP relationship for the Kawasaki-, Tanaka-, and INTERSALT-estimated urine sodium (Figure 2). Inserting the sex-specific mean constant sodium in the formulas resulted in a loss of the J-shaped appearances of all the formula-estimated sodium excretion and DBP relationships (Figure 3).

### Sensitivity Analyses

The J-shaped urine sodium-SBP and sodium-DBP relationships became attenuated in the restricted cubic spline plots when samples were excluded that did not have a creatinine index ≥0.7 (n=6308 person-visits included; Figure S3). The J-shaped relationships were completely lost when samples were excluded if the mCER was not within 15% of the Kawasaki-predicted urine creatinine excretion (n=1608 person-visits included; Figure S4). In cohort 1, we compared the estimated 24-hour urine sodium from spot urine samples, estimates from 24-hour urine sodium concentration, and measured sodium in 24-hour urine. Each estimate of 24-hour urine sodium had a differing association with BP compared with m-24hUNa. There was a similar shaped urine sodium-SBP relationship and similar shaped sodium-DBP relationship for 24-hour urine sodium versus spot urine sodium concentrations inserted into the Kawasaki and Tanaka formulas (Figure S5). However, the urine sodium-SBP and sodium-DBP relationships were markedly different for the 24-hour urine sodium versus the spot urine sodium concentrations inserted into the INTERSALT formula (Figure S5).

## Discussion

We found a positive steep linear association between m-24hUNa excretion with SBP and DBP up to the median value (≈145 mmol/24 hours), followed by a positive but less steep linear association at higher sodium excretion. In contrast, the relationships between formula-estimated 24-hour urine sodium excretion with SBP and DBP were negative up to the estimated median 24-hour urine sodium excretion values. The estimated 24-hour urinary sodium by the 3 formulas (Kawasaki, Tanaka, and INTERSALT) all showed a J-shaped relationship with SBP and DBP. The altered associations between the formula-estimated 24-hour urine sodium and BP are specifically due to inherent properties of these formulas because the concentrations of sodium and creatinine (and potassium for the INTERSALT equations) were derived from 24-hour urine samples, thus excluding errors that might be associated with the collection of spot urine samples. Notably, the formula-estimated urine sodium still had associations with SBP when a sex-specific constant sodium concentration was inserted in the formulas rather than the measured sodium concentration, indicating the formulas are associated with BP, independent of sodium. A previous investigation from the Trials of Hypertension Prevention found that the same formulas also altered the association of estimated sodium excretion to total mortality.^17,26^ To the best of our knowledge, our study is the first to document that sodium-estimating equations alter the sodium-BP association.

Our restricted analyses (sensitivity analyses) for person-visits of creatinine index ≥0.7 attenuated the J-shaped relationship between formula-predicted urine sodium excretion and SBP, and restricted analyses among person-visits of mCER within 15% of Kawasaki-predicted urine creatinine excretion did not have a J-shaped relationship. These methods were developed for assessing the completeness of 24-hour urine sample collections. The creatinine index ≥0.7 method excludes samples with a low measured urine creatinine, and the more restrictive mCER within 15% of Kawasaki-predicted urine creatinine excretion method excludes urine samples with both low and high measured urine creatinine. Hence, the exclusion and inclusion of urine samples with outlying mCER values also interacts with the various formulas to alter the association of sodium excretion and BP—a unique contribution of our findings. Our findings also emphasize the need to take great care to ensure 24-hour urine samples are complete in analyses associating urine sodium with health outcomes. Although spot urine samples are not influenced by completeness, there could be substantial variability of individuals’ urine creatinine concentrations based on age, sex, muscle mass, and protein intake.^12,32,33^ One of the principal assumptions for constructing KAWASAKI and TANAKA formulas was that the predicted urinary creatinine excretion is equivalent to the mCER.^15,16^ This assumption is not valid in our study population. Only 16% (1608/10 034) of person-visits had mCER within 15% of Kawasaki-predicted urine creatinine excretion in our study population. These findings also highlight that our results’ generalizability is linked to urine creatinine variability at the population level. Our results are likely to be generalizable for populations with similar characteristics such as age and sex distribution, ethnicity, sodium intake, dietary habits, muscle mass, and physical activity. The urine sodium measurement error due to spot urine formulas may be more prominent in conditions with altered urine creatinine levels, such as populations with high muscle mass, renal impairment, or muscle breakdown. Our study had greater ability to observe an altered association between estimated 24-hour urine sodium and BP compared with studies with more homogeneous participants.

Consistent with previous research, we found all the formulas had biased estimates of 24-hour sodium excretion and differing biases for estimated sodium excretion at higher or lower sodium excretion levels. We are not aware of any validation study for these 3 equations among the Bangladeshi population. Nevertheless, the spot urine formulas have been used in several studies from Bangladesh for assessing the mean sodium excretion of the population^34^ and evaluating the association between sodium excretion and BP.^35,36^ Based on our results and a meta-analysis, the Bangladesh studies are likely to have underestimated the 24-hour sodium excretion of the communities in coastal Bangladesh. Sodium measurement from spot urine samples may also provide different results than 24-hour urine sodium due to circadian and meal-related variation in sodium excretion.^37,38^

We did not find any association between 24-hour measured urine sodium and DBP. High-quality evidence from randomized controlled trials has found lower sodium intake is associated with a lower DBP.^39,40^ The lack of an association between sodium intake and DBP in our study is likely related to the limitations of the cohort study design. However, as for SBP, the relationship between urinary sodium excretion and DBP was altered by the formula-estimated urine sodium. This indicates that there is the same inherent problem within the formulas for SBP and DBP.

One important methodological criticism of inserting 24-hour urine sodium and creatinine concentrations in the Kawasaki formula is that the Kawasaki formula was developed specifically for second-morning urine excretion samples.^15^ Nevertheless, we found a similar urine sodium-SBP relationship for the Kawasaki and Tanaka formulas when 24-hour urine versus second-morning spot sodium was used in the second sensitivity analysis, which supports the use of 24-hour urine sodium and creatinine values in these 2 formulas. The INTERSALT formula was not developed from second-morning spot urine samples but was developed from casual urine samples (eg, random spot urine samples).

Our analyses have both strengths and important limitations. The study had >10 000 person-visit data, providing high statistical power to associate sodium excretion with BP. Many participants from southwest coastal communities in Bangladesh were followed up in both the second and third cohort studies, 5 to 12×, over a period of 2.5 years. Since we used individual-, household-, and community-level random intercepts to account for data clustering, our estimates will not be biased by many unmeasured time-invariant confounders at the individual, household, and community levels.

The Kawasaki and Tanaka formulas were developed in Japanese populations, whereas the INTERSALT formula was developed using data from the European and North American populations. Therefore, the utility of these formulas may vary across populations. Our 24-hour urine sample collections were also likely affected by both over- and undercollection.^12,41^ Para-aminobenzoic acid has been recommended to assess the completeness of 24-hour urine collection studies^42^ but was not assessed in our study. BP has a diurnal variation, with morning BP usually higher than that in the afternoon.^43^ Since we did not collect the exact time of BP measurement, we could not control for diurnal variation of BP.

Currently, it is recommended that a minimum of 3 nonconsecutive 24-hour urines be used to assess an individual’s usual dietary sodium intake in studies associating sodium intake to health outcomes.^42^ Based on our study findings, formula-estimated 24-hour sodium excretion should not be used to examine the relationship between sodium excretion and health outcomes. Studies that use spot urine samples and relate them to health outcomes are likely to produce false associations generated by age, sex, weight, and creatinine. Such spurious findings are likely to increase the debate about health outcomes at low sodium intake. While our findings indicate the role of the population’s urine creatinine variability on the urine sodium measurement error, further research is warranted to better understand urine creatinine’s role in studies linking sodium intake and health outcomes in diverse populations. Databases with spot urine sodium equations that have been used to associate dietary sodium to health outcomes should be reanalyzed using constant sodium values to assess the role of the formulas in producing spurious results that are independent of sodium intake.

## Perspectives

Our results suggested that spot urine formulas lead to spurious J-shaped associations between the formula-predicted daily sodium intake and SBP. Our results emphasize the use of 24-hour urine sodium as the proxy of daily sodium intake in epidemiological studies that associate sodium intake with health outcomes.

## Acknowledgments

We acknowledge with gratitude the commitment of the Wellcome Trust, United Kingdom, for supporting the research. We are grateful to the participants in the study for their support and cooperation. The International Centre for Diarrhoeal Disease Research, Bangladesh is also grateful to the governments of Bangladesh, Canada, Sweden, and the United Kingdom for providing core and unrestricted support. We are grateful to our colleagues at the University of Dhaka and the United Nations International Children’s Emergency Fund, Bangladesh, who helped to implement the studies.

## Sources of Funding

This research was funded by the Wellcome Trust, United Kingdom, through an award under the Our Planet, Our Health Programme (grant No. 106871/Z/15/Z). A.M. Naser’s time was partly supported by the National Heart, Lung, and Blood Institute–funded T32 training grant (grant No. T32 HL130025).

## Disclosures

F.J. He is an unpaid member of Action on Salt and World Action on Salt and Health (WASH). N.R.C. Campbell is an unpaid member of WASH. The other authors report no conflicts.

## Supplementary Material



## Novelty and Significance

### What Is New?

- This study is the first documentation of the J-shaped association between spot urine formula–predicted urine sodium and blood pressure. Previous studies have reported the J-shaped association between spot urine formula–predicted urine sodium and cardiovascular disease events. Second, this study highlights that the use of urine creatinine concentration in the spot urine formula may be one reason for the spurious J-shaped association between spot urine formula–predicted urine sodium and blood pressure.

### What Is Relevant?

- Spot urine formula–predicted urine sodium should not be used in epidemiological studies to link sodium intake and health outcomes.

### Summary

There is a positive dose-response relationship between sodium intake and health outcomes. Our findings support the World Health Organization recommendation of low sodium–containing diet promotion in the population.
