# Supplementary figures and images for: Spot Urine Formulas to Estimate 24-Hour Urinary Sodium Excretion Alter the Dietary Sodium and Blood Pressure Relationship
Source: Hypertension. 2021 Apr 5;77(6):2127–37. doi: 10.1161/HYPERTENSIONAHA.120.16651 (PMC8115426; doi:10.1161/HYPERTENSIONAHA.120.16651)

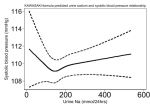

Supplement: Supplementary file 1 [file hyp-77-2127-s001.jpg]
