# Supplementary material for: Spot Urine Formulas to Estimate 24-Hour Urinary Sodium Excretion Alter the Dietary Sodium and Blood Pressure Relationship
Source: Hypertension. 2021 Apr 5;77(6):2127–37. doi: 10.1161/HYPERTENSIONAHA.120.16651 (PMC8115426; doi:10.1161/HYPERTENSIONAHA.120.16651)
Supplement: Supplementary file 2 [file hyp-77-2127-s002.doc]

**Spot-urine formulas to estimate 24-hour urinary sodium excretion alter the dietary sodium and blood pressure relationship**

**Short title: Urine sodium excretion and blood pressure**

Abu Mohd Naser^1,2*^, Feng J. He^3^, Mahbubur Rahman^4^, Norm R.C. Campbell^5^

^1^ Emory Global Diabetes Research Center, Hubert Department of Global Health, Rollins School of Public Health, Emory University, Atlanta, Georgia, USA

^2^ Department of Epidemiology, Rollins School of Public Health, Emory University, Atlanta, Georgia, USA

^3^Centre for Environmental and Preventive Medicine, Wolfson Institute of Preventive Medicine, Barts and The London School of Medicine and Dentistry, Queen Mary University of London, United Kingdom

^4^ Environmental Interventions Unit, Infectious Diseases Division, International Center for Diarrhoeal Disease Research, Bangladesh (icddr,b), Dhaka-1212, Bangladesh

^5^ Department of Medicine, O’Brien Institute of Public Health, Libin Cardiovascular Institute of Alberta at the University of Calgary, Canada

*Corresponding author:

Abu Mohd Naser, MBBS, PhD

Emory Global Diabetes Research Center, Hubert Department of Global Health, Rollins School of Public Health, Emory University, Atlanta, Georgia, USA

Rollins School of Public Health | Emory University

2nd floor, CNR building, Room # 2030E

Atlanta, GA 30322, USA

email: atitu@emory.edu

Table S1: Kawasaki, Tanaka, and INTERSALT equation

| Measurement | Formula |
| --- | --- |
| KAWASAKI formula for 24-hour Na, mmol/day | 16.3× (Na (mmol/L)/Cr (mg/dL) × estimated 24UCr (mg/day)) ^0.5  Where, estimated 24UCr for male =  (-12.63×age+15.12×weight+7.39× height-79.90)  estimated 24UCr for female = (-4.72×age+8.58× weight+5.09× height-74.50) |
| Tanaka formula for 24-hour Na, mmol/day | 21.98× [(spot Na(mmol/L)/ spot Cr (mg/dL) × 10 × estimated 24UCr] ^ (0.392)  Where, estimated 24UCr = (-2.04 × age+14.89 × weight+16.14 × height-2244.45) |
|
| INTERSALT formula for 24-hour Na, mmol/day | Male: ((25.46+0.46× spot Na (mmol/L))-2.75× spot Cr (mmol/L) -0.13× spot K (mmol/L) +4.10×bmi+0.26×age)  Female: ((5.07+0.34× spot Na (mmol/L))-2.16× spot Cr (mmol/L) -0.09× spot K (mmol/L) +2.39×bmi+2.35×age-0.03×age^2^) |


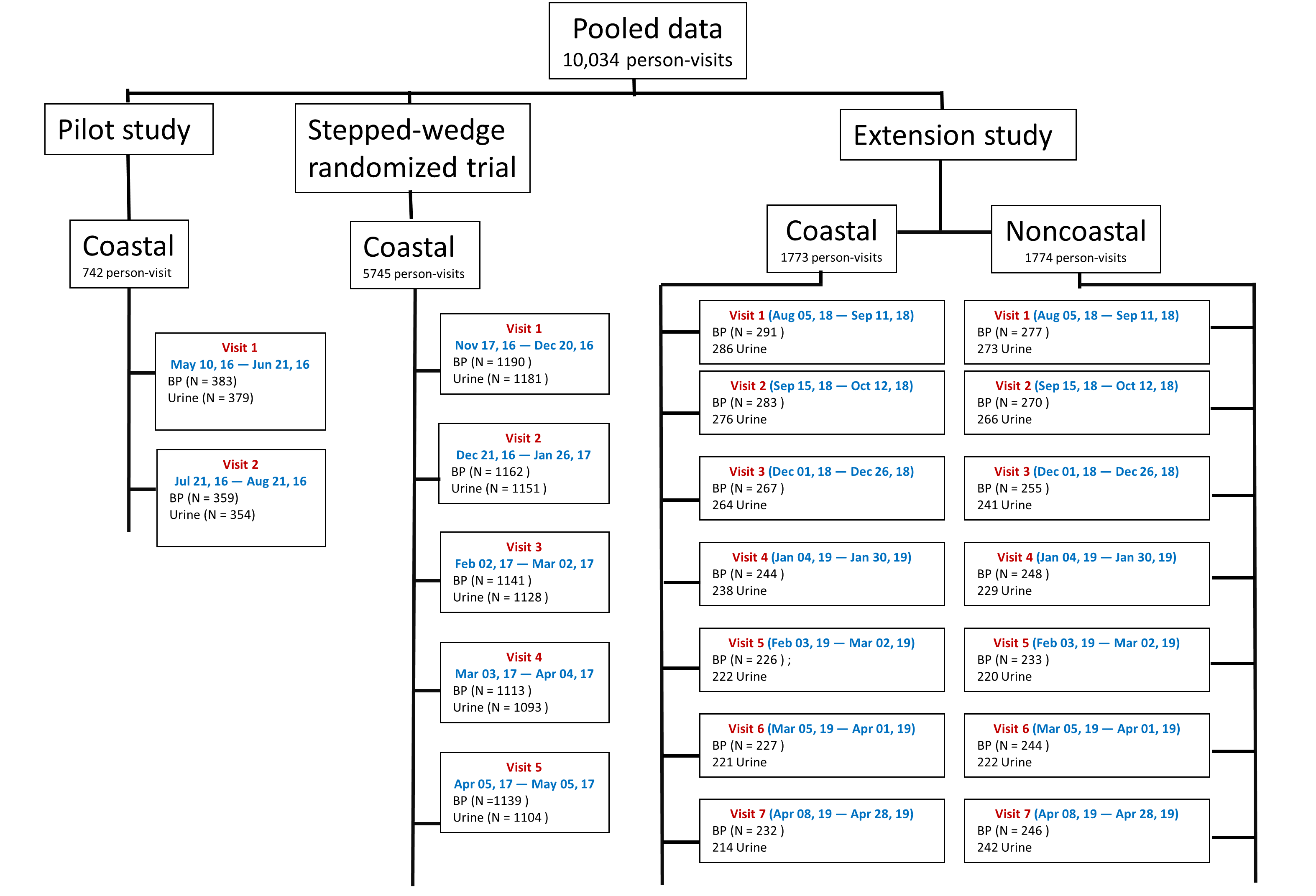


**Figure S1: Sources of data**

**
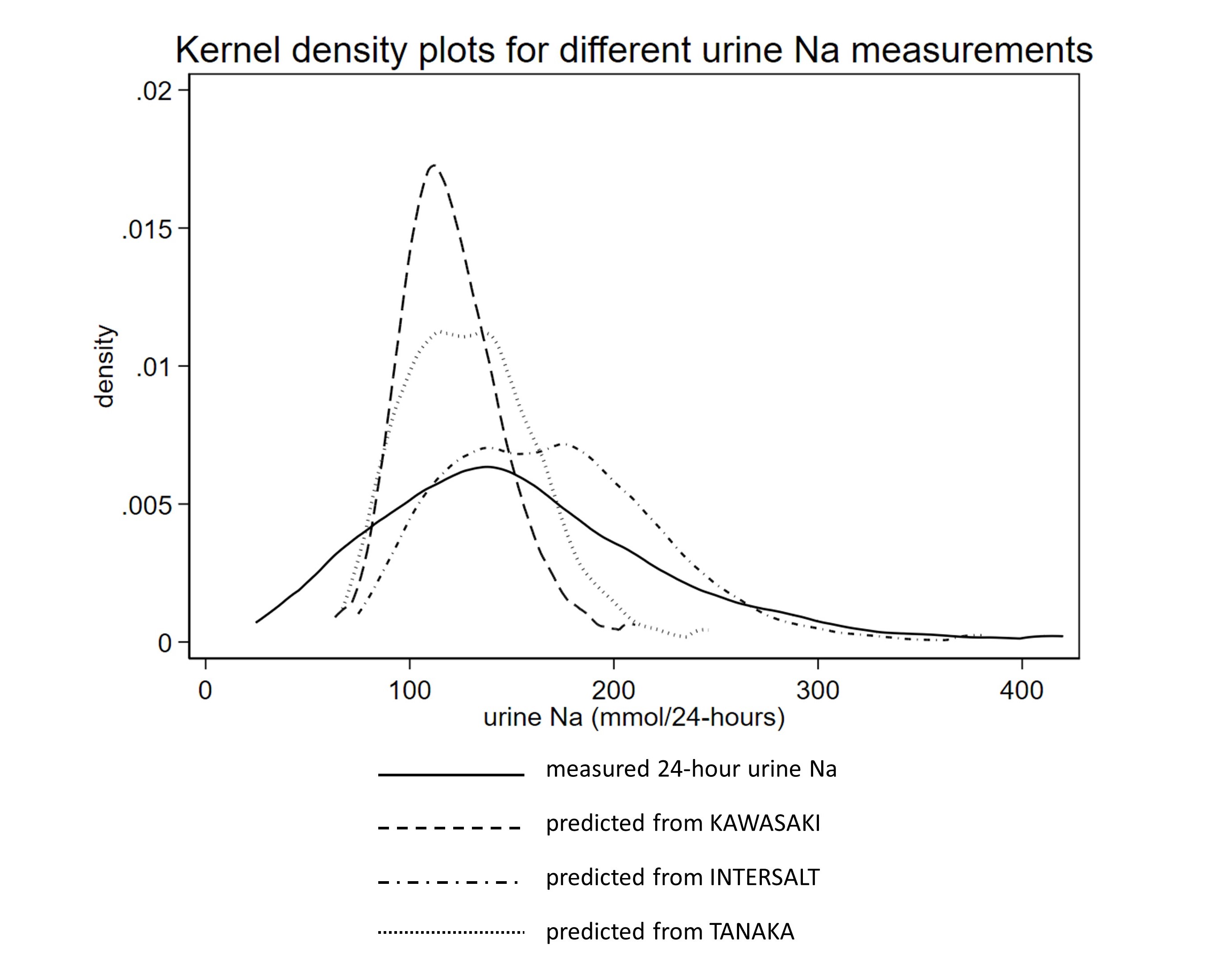
**

**Figure S2: Kernel density plots for different urine sodium (Na) measurements**

**
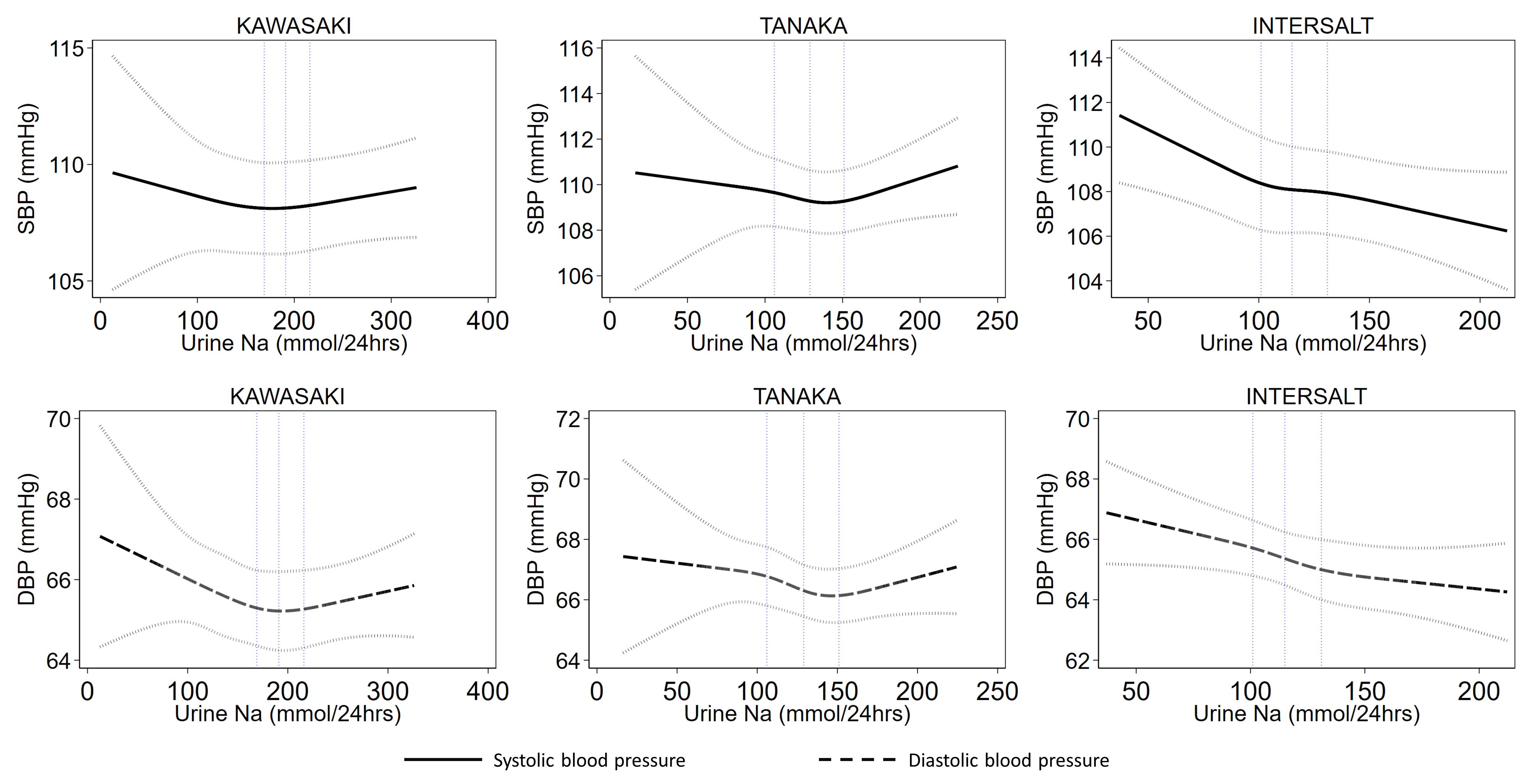
**

Figure S3: Restricted cubic spline plots and 95% confidence interval (dotted lines) for sodium excretion and blood pressure relationship using different methods of estimating urinary sodium excretion from person-visits included by creatinine index ≥0.7 and no self-reported missing voids. Models were adjusted for age, sex, BMI, smoking status, physical activity, sleep, alcohol consumption, religion, and household wealth. Vertical dotted blue lines indicate 25^th^, 50^th^, 75^th^percentile distribution of urine sodium excretion.

**
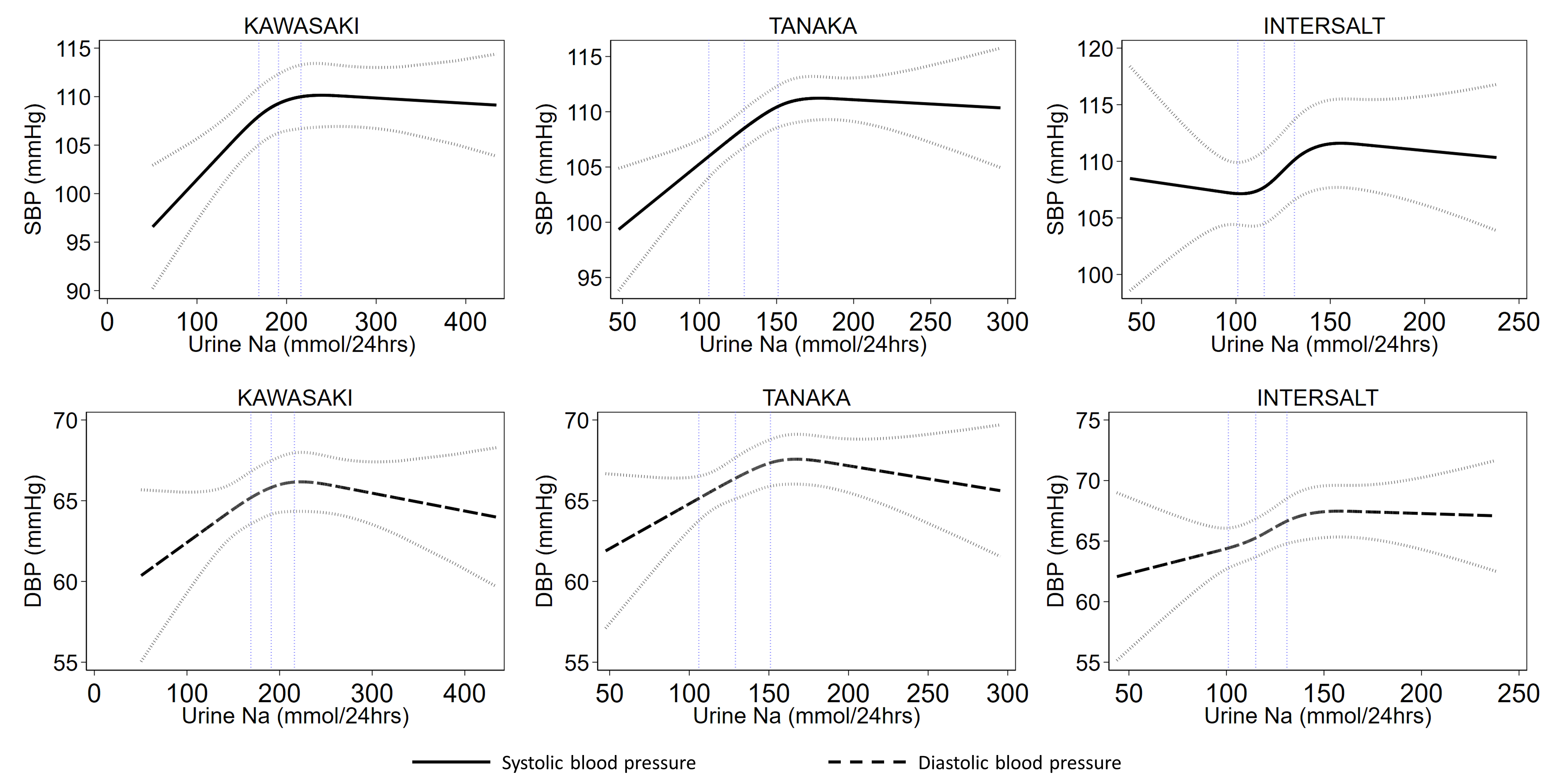
**

Figure S4: Restricted cubic spline plots and 95% confidence interval (dotted lines) for sodium excretion and blood pressure relationship using different methods of estimating urinary sodium excretion from person-visits included by mCER with 15% of KAWASAKI predicted urine creatinine excretion and no self-reported missing voids. Models were adjusted for age, sex, BMI, smoking status, physical activity, sleep, alcohol consumption, religion, and household wealth. Vertical dotted blue lines indicate 25^th^, 50^th^, 75^th^percentile distribution of urine sodium excretion.

**
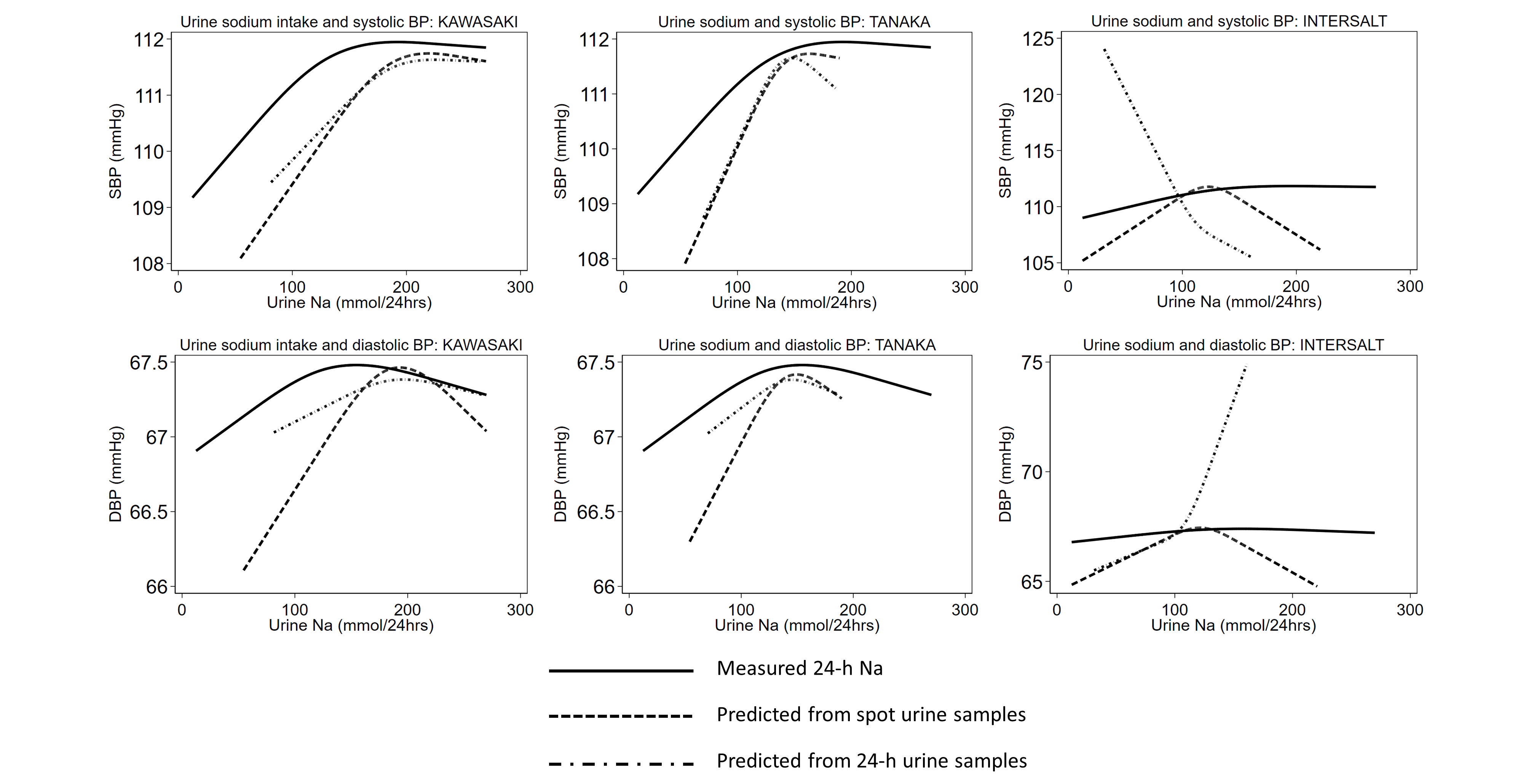
**

Figure S5: Restricted cubic spline plots for urine sodium excretion and blood pressure relationship for three types of urine sodium from cohort 1 study: 1) measured 24-hour urine sodium, 2) formula-estimated urine sodium when 24-hour urine sodium concentrations were inserted into the formulas, and 3) formula-estimated urine sodium when 2^nd^ morning spot urine sodium concentrations were inserted into the formulas. Models were adjusted for age, sex, BMI, smoking status, physical activity, sleep, alcohol consumption, religion, and household wealth.
